# Life & Advice from the Giants in Cardiac Surgery: Giving & Receiving; Leading & Following

**DOI:** 10.21470/1678-9741-2024-0995

**Published:** 2024-10-14

**Authors:** Teresa M. Kieser

**Affiliations:** 1 Cumming School of Medicine, University of Calgary, Libin Cardiovascular Institute, Calgary, Alberta, Canada

## Teresa M. Kieser

“Dear Colleagues, Ladies and Gentlemen,

I am no giant. I think of myself as an ordinary every-day cardiac surgeon who does a lot of arterial grafts, one graft at a time. I have not done enough yet to be considered a giant but thank you EACTS for this great honour and for regarding me as such.”

(Teresa M. Kieser)

This text will address three topics related to Dr. Kieser's career: 1) the highlight of her career, 2) the most recent development in cardiac surgery in her time, and 3) how young cardiac surgeons might prepare for their future-by giving examples from her nearly 37-year career, as of Jan 1, 2025. Dr. Kieser suggests that the following 15 points can be considered guiding principles for any career.

### 1. Do not listen to “nay-sayers”; they either do not understand or know you or may not have your conviction of purpose

In her 4^th^ year of medical school, Dr. Kieser informed her staff surgeon that she wanted to be a general surgeon, to which he replied, “But you are a woman, who will send you business?” The referrals did take a while but eventually started flowing steadily after about 13 years in practice, at a time when bilateral internal mammary artery (BIMA) grafting would begin to show its promise. In a way, he was right.

### 2. Be firm in your convictions

Dr. Kieser spent two years of military service working with cardiologist Dr. Gerald M. FitzGibbon. When she asked him for a reference letter for General Surgery, he expressed doubts, saying he did not believe she was “surgical material” and did not envision her going to the operating room or talking about surgery. Instead, he suggested she might be better suited for Internal Medicine or Cardiology. This was surprising, as at the very beginning Dr. FitzGibbon had promised to help her to obtain a third year of surgical residency if she worked two years for him. Feeling incensed, Dr. Kieser pounded his desk with her fist and reminded him of his promise. Dr. FitzGibbon sat back with a smile and said, “Well, this is a butterfly of different colours”. As a result, this year of surgery counting as a third and final year of military service came to pass.

### 3. Be open to change

Although General surgery was Dr. Kieser’s first choice, her third military year in surgery included three months with Dr. WJ Keon at the now Ottawa Heart Institute. At the start of this rotation, her goal was still to become a general surgeon, but by the end of the three months, her focus shifted to cardiac surgery: the nurses in the Cardiovascular Intensive Care Unit (CVICU) loved her-not only she could treat atrial fibrillation and heart failure, but she could also quickly open a chest. Dr. Keon even prophesied that, like him, she would one day open her own cardiac surgery unit.

### 4. Do not follow the crowd

The invaluable mentorship of Drs. FitzGibbon and Keon in the first three of Dr. Kieser’s 12 years of training laid the groundwork for her entire career. Having looked at countless diseased vein grafts with Dr. FitzGibbon and hearing Dr. Keon telling her in 1983 “Do arterial grafts; they are the future” (three years before Floyd Loop’s 1986 paper extolling the virtues of one internal mammary artery - IMA)^[1]^, she embarked on a career of BIMA grafting. This approach was unconventional at the time. Dr. Kieser recalls an anaesthetist in the early 1990s leaning over the OR drape and questioning her decision to use two IMAs on a patient with diabetes. She responded by explaining that patients with diabetes needed BIMA grafting more than anyone else.

### 5. Do not fear going to the extraordinary

Dr. Kieser's first job after completing her training was to commence the University of Calgary Cardiovascular and Thoracic Surgery Program at Foothills Medical Centre in 1988. As she hired personnel, including head nurses and perfusionists, and wrote protocols for preoperative, operative, and postoperative care, she recognized the inherent risk in assembling a team that had never worked together before, with members coming from various parts of Canada. When the VP of Development initially reacted negatively to the idea of “putting a pig on pump” for practice, she simply asked him, “Would you prefer we find out the glitches on a human being?” As a result, “pigs on pump” prevailed, revealing three issues that, while not major, were important to require correction.

### 6. Keep focused no matter what befalls you

During the 19 months when Adrienne, Dr. Kieser’s younger daughter, battled a brutal T-cell leukemia from ages 3 to 5, Dr. Kieser’s surgical results were never better. Her husband, Jean, worked from home in his commercial realty job while looking after their older daughter, Alexandra, who was 7 to 9 years old during that period. Dr. Kieser spent her days working at the hospital and her nights with Adrienne whenever she was admitted, which was frequently. Despite the profound challenge of losing Adrienne, Dr. Kieser reflects on this period with the belief that her family was supported by their faith, often recalling the poem "Footprints in the Sand" as a reminder of how they were carried through.

### 7. Follow your heart, not your mind

**Fig. 1:**
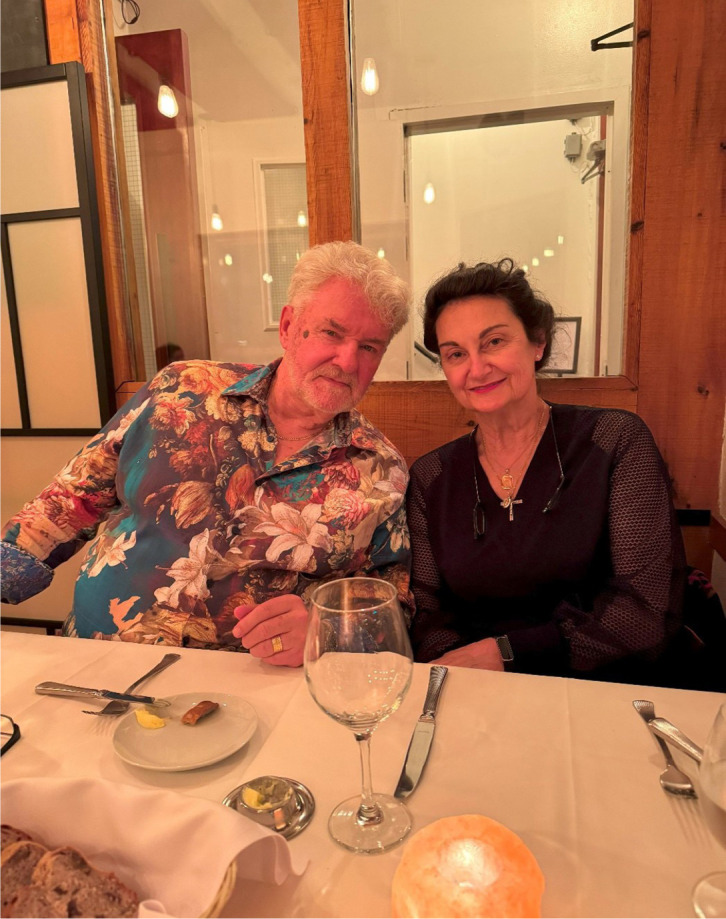
Dr. Kieser and her husband, Jean.

Dr. Kieser recalls that she and Jean (Figure 1) fell in love on their second date, and he has been her rock, closest friend, and staunch advocate for 38 years. Jean has helped her stay grounded and has gently offered insights, even helping her to understand why her male colleagues behaved the way they did and how she could be more subtle. His wisdom has been invaluable. When they lost their daughter Adrienne and were deep in grief, he simply said one day, “Would we ever have been without her?” And when Dr. Kieser worried over a lost patient, he would say, “If you saved everyone, you wouldn’t be a doctor”. She feels that having a soul mate in life is a gift beyond compare.

### 8. Keep your cards close to your chest

Dr. Kieser often reflects on the “20-30-50 rule” shared with her by her brother-in-law, Francois. According to his advice, “20% of the people around you love you and want you to succeed, they are your family, your close friends; 30% are not your friends and want to see you fail and the other 50%… are watching to see which way you fall”.

### 9. Be open to trying new things

One day, the chief perfusionist approached Dr. Kieser and asked if she would you like to try a new machine for checking bypass grafts, which had been provided for free with the valves from Medtronic. She agreed. This was on April 29, 2004, and 18 years later, Dr. Kieser has witnessed the evolution of intraoperative graft assessment for coronary surgery. As coronary surgery is still half of cardiac surgical practice in many places, she considers this development to be the most significant advancement in cardiac surgery during her career.

### 10. Never stop learning: never stop striving to make things better for your patients

A young PhD student of Professor Pieter Kappetein, Stuart Head, suggested to Dr. Kieser that she could pursue her PhD. She responded by saying, “Stuart, I am too old to do rat surgery in laboratories”. However, Stuart reassured her, pointing out that all her work was centered one a single theme-improving the bypass operation. During a car ride to Lake Louise, Professor Kappetein turned them and said, “Yes, you can, and I will be your promoter”. As a result, on October 8, 2015-almost nine years ago-Dr. Kieser defended her PhD^[2]^, with Stuart Head and Ruben Osnabrugge serving as her two paranymphs. Her experience reinforced the belief that one is never too old to learn.

### 11. If I had to pick the highlight of my career so far, it has been travelling all over the world teaching young and eager surgical minds

**Fig. 2:**
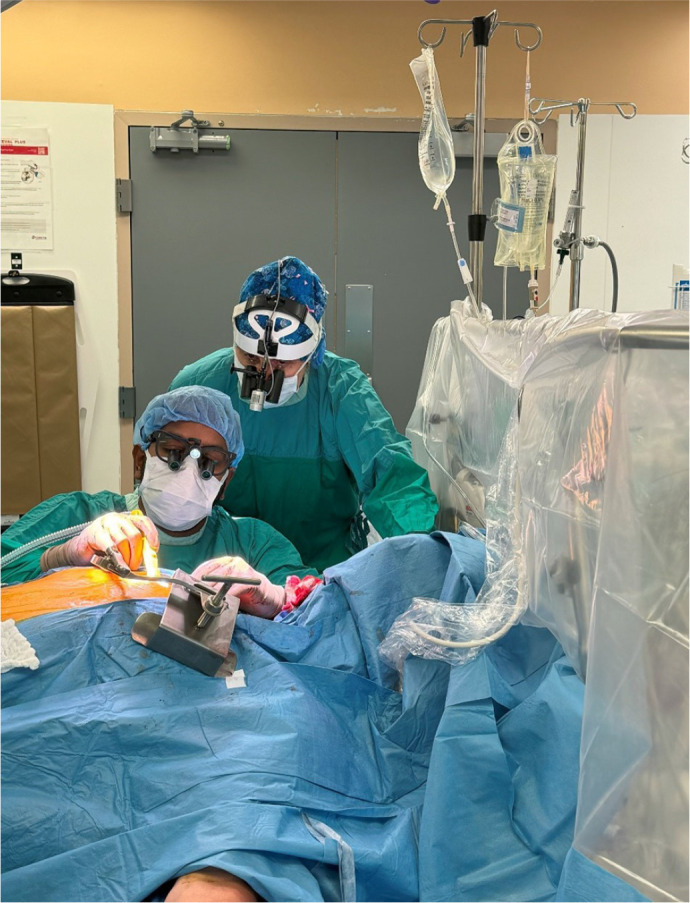
Dr. Kieser teaching the IMA harvesting procedure.

According to Dr. Kieser, teaching is always a two-way street; in fact, she finds that you always learn much more than you give (Figure 2).

### 12. However, if hard work is all that was needed, it would have been easy…

In June 2003, during a conversation with Professor Alfieri at a meeting in A Coruña, he asked Dr. Kieser what it was like to perform so many arterial grafts. She replied by saying that it pleased the people she worked for-the cardiologists appreciated the grafts' longevity, and patients were happy because their legs were not being cut. However, it also had its downsides, as it frustrated her team due to the longer hours required and annoyed her colleagues, who thought she was grandstanding. After hearing this, Professor Alfieri said, “Teresa, you are very interesting to talk to”.

### 13. Follow your own path

Dr. Kieser acknowledges that pursuing an unexplored path may bring challenges and suffering. However, if your goal is to serve others without seeking reward, and to genuinely improve their lives, you will not fail. By honoring the gifts you have been given and “bearing fruit with patient endurance” (Luke 8, 4-15), you will be able to follow Shakespeare’s advice: “This above all, to thine own self be true”.

### 14. St Paul said: “What do you have that you were not given? Then why do you boast as though it were not a gift?” He also said: “By the grace of God, I am what I am”

Dr. Kieser believes she owes a great debt to God and to all her fellow women and men.

### 15. And finally, never, never give up. Thank you

Dr. Kieser emphasized the importance of perseverance, urging others to never give up.
